# Identification of Parasitic Communities within European Ticks Using Next-Generation Sequencing

**DOI:** 10.1371/journal.pntd.0002753

**Published:** 2014-03-27

**Authors:** Sarah Bonnet, Lorraine Michelet, Sara Moutailler, Justine Cheval, Charles Hébert, Muriel Vayssier-Taussat, Marc Eloit

**Affiliations:** 1 USC INRA Bartonella-tiques, UMR BIPAR ENVA-ANSES, Maisons-Alfort, France; 2 PathoQuest SAS, Paris, France; 3 Ecole Nationale Vétérinaire d'Alfort, UMR 1161 Virologie ENVA, INRA, ANSES, Maisons-Alfort, France; 4 Institut Pasteur, Laboratory of Pathogen Discovery, Paris, France; University of Texas Medical Branch, United States of America

## Abstract

**Background:**

Risk assessment of tick-borne and zoonotic disease emergence necessitates sound knowledge of the particular microorganisms circulating within the communities of these major vectors. Assessment of pathogens carried by wild ticks must be performed without *a priori*, to allow for the detection of new or unexpected agents.

**Methodology/Principal Findings:**

We evaluated the potential of Next-Generation Sequencing techniques (NGS) to produce an inventory of parasites carried by questing ticks. Sequences corresponding to parasites from two distinct genera were recovered in *Ixodes ricinus* ticks collected in Eastern France: *Babesia* spp. and *Theileria* spp. Four *Babesia* species were identified, three of which were zoonotic: *B. divergens*, *Babesia* sp. EU1 and *B. microti*; and one which infects cattle, *B. major*. This is the first time that these last two species have been identified in France. This approach also identified new sequences corresponding to as-yet unknown organisms similar to tropical *Theileria* species.

**Conclusions/Significance:**

Our findings demonstrate the capability of NGS to produce an inventory of live tick-borne parasites, which could potentially be transmitted by the ticks, and uncovers unexpected parasites in Western Europe.

## Author Summary

Diseases transmitted by ticks have diverse etiology (viral, bacterial, parasitic) and are responsible for high morbidity and mortality rates around the world, both in humans and animals. The emergence or re-emergence of tick-borne diseases is increasingly becoming a problem as the geographical distribution of several tick species is expanding, as well as the numbers of potential or known tick-borne pathogens are constantly evolving. It is thus necessary to know which microorganisms circulate within communities of this major vector to ensure adequate epidemiological surveillance. In this study, we evaluated the potential of Next-Generation Sequencing techniques (NGS) to produce, without *a priori*, an inventory of both predicted and non-expected parasites carried by *Ixodes ricinus*, the most prevalent human biting tick in France. Our findings suggest that NGS strategies could be used to produce an inventory of live parasites residing in ticks from a selected area, thereby expanding our knowledge base of tick-associated parasites.

## Introduction

Due to the combination of increased human and animal movement, socio-economic and environmental changes, as well as the complex interactions between reservoirs, pathogens, and human populations, more emerging diseases are being identified and the epidemiology of ancient diseases is changing, particularly that of vector-borne diseases [1]. After mosquitoes, ticks are the most common worldwide vector that can affect both humans and animals, and can transmit the highest variety of pathogens, including viruses, bacteria and parasites. Of these parasites, *Babesia* sp. or *Theileria* sp. are two well-known parasites responsible for several diseases that impact both human and animal health worldwide [2], [3]. *Ixodes ricinus* is the most prevalent tick in Europe and the vector for several bacterial and viral pathogens [4], as well as three parasites: *B. divergens*, *B. microti* [4] and *Babesia* sp. EU1 [5], [6]. To date, no other parasites have been reported to be transmitted by this tick species, even though these ticks feed on a very large spectrum of hosts potentially infected by several parasite species. However, the list of potential or known tick-borne pathogens is constantly evolving, and emergence or re-emergence of tick-borne diseases leads to the development of unknown health risks [4]. Therefore there is a real concern that tick-borne diseases will appear in areas previously free of such diseases, consequently new studies are required to catalog those parasitic communities hosted by, and potentially transmitted by ticks.

Traditionally, identification of microorganisms has relied on their cultivation in artificial environments, but it has become evident that ticks harbor a variety of microbes that may have obligate intracellular life histories and/or require highly specific medium for their cultivation, resulting in the impossibility of successfully culturing some microorganisms, especially parasites. Thus, the identification of tick-borne parasites increasingly relies on molecular detection approaches. Classically, pathogen detection in ticks is performed by PCR with specific primers. These are designed to amplify conserved microbial sequences in a predefined list of pathogens known to be transmitted by the specific collected tick species, in the specific geographical area of collection. However, this method is not at all suited to detect new or unexpected pathogens [7], [8]. In addition, because of the relative paucity of available sequence data for tick-borne parasites, most of these techniques rely on the amplification of the 18S genes which are well conserved among parasites, implying an additional sequencing step in order to identify them at the species level. Finally, the amount of available DNA in a tick sample limits such detection to a limited number of PCR tests. Consequently, a detailed inventory of pathogenic agents carried by ticks must be carried out without *a priori*, necessitating novel approaches. Recently, the metagenomic profiles of the bacterial communities associated with the *Ixodes ricinus* tick have been assessed using Next Generation Sequencing (NGS) methods, which permits the characterization of the entire tick microbiome based on 16S rRNA sequencing [9], [10]. However, such an approach does not allow identification of the bacteria at the species level, which is absolutely essential when distinguishing symbionts and commensals from the pathogenic bacteria carried by the ticks. To avoid this problem, we recently and successfully used a similar approach, but which sequenced the entire transcriptome of ticks, generating an in-depth picture of bacteria carried by *Ixodes ricinus* from Eastern France, and that led to the identification of both known and unexpected tick-borne bacteria [11]. In this study NGS with a similar protocol was used to produce an inventory of known and unexpected parasites carried by *I. ricinus* in the same area of Eastern France.

## Materials and Methods

### Study area and tick collection

A total of 1478 *I. ricinus* questing nymphs were collected by flagging in three forested areas of Eastern France (Alsace Department): Murbach (47°55′05″N, 7°8′46″E), Hohbuhl (48°27′33″N, 7°17′22″E) and Wasselonne (48°38′09″N, 7°21′45″E), a region with abundant ticks and a concomitant high risk of disease transmission. Ticks were pooled into groups of 15 individuals and crushed in 300 µl of Dulbecco's MEM (DMEM) medium supplemented with 10% fetal bovine serum. A pool of 15 *I. ricinus* nymphs from our pathogen-free colony was treated equivalently and used as a reference as previously described [11]. This control colony originated from female ticks collected in Murbach and was reared as previously described [12].

### High throughput sequencing and data analysis

High throughput sequencing of tick pool samples was performed as previously described [11]. Briefly, total RNA, which indicates the occurrence of viable and replicating microorganisms, and total DNA, for specific real-time PCR, were separately extracted. Wild and pathogen-free RNA samples were sequenced to a depth of 100 million and 62 million for 101 bp paired-end reads respectively. As there is no publicly available *I. ricinus* reference genome, we removed those sequences corresponding to the ticks themselves, or to symbiotic or commensal bacteria naturally found in ticks, by subtracting sequences homologous to sequences from the pathogen-free reference sample using the SOAP2 aligner tool. Finally, 7 787 463 remaining reads out of 70 396 392 reads initially obtained from wild ticks, were used for *de novo* assembly, producing 174 841 contigs. Contigs were then assigned the closest known taxonomy according to their identity percentage (Blast search option of the National Center for Biotechnology Information, www.ncbi.nlm.nih.gov/BLAST), and distant alignments were not considered. Of the assigned reads, 6.65% of the cDNA derived sequences were of a parasitic origin, corresponding to 0.73% of the reads obtained from whole wild ticks. Among these sequences, contigs of significant interest were selected based on at least one of the following criteria 1) an identity percentage >95% with a particular parasite species, 2) known to be responsible for human or/and animal disease and 3) a high read number.

### Confirmation of parasite targets with quantitative PCR

Real-time PCR was performed on DNA extracted from each pool of ticks to confirm taxonomic species assignment of NGS-derived contigs. Amplification was performed as previously described [11] and the primers newly designed for this study, based on the 18S rDNA, *hsp*70 and CCTeta sequences present in GenBank, are presented in Table 1. *Babesia* and *Theileria* DNA used for positive controls were kindly provided by Huseyin Bilgic, Faculty of Vet.Med, Turkey; Laurence Malandrin, ONIRIS, France; Emmanuel Cornillot, Montpellier University, France.

**Table 1:** Primers and probes designed and used for the detection of *Babesia* species and *Theileria* species via quantitative PCR.

Pathogen	Gene target	Primer or probe	Sequence (5′→3′)	Amplicon size (bp)
*Babesia divergens*	hsp70	Bdi_F	CTCATTGGTGACGCCGCTA	83
		Bdi_R	CTCCTCCCGATAAGCCTCTT	
		Bdi_P	AGAACCAGGAGGCCCGTAACCCAGA	
*Babesia* sp.EU1	18S rRNA	BEU1_F	GCGCGCTACACTGATGCATT	91
		BEU1_R	CAAAAATCAATCCCCGTCACG	
		BEU1_P	CATCGAGTTTAATCCTGTCCCGAAAGG	
*Babesia microti*	CCTeta	Bmi_F	ACAATGGATTTTCCCCAGCAAAA	145
		Bmi_R	GCGACATTTCGGCAACTTATATA	
		Bmi_P	TACTCTGGTGCAATGAGCGTATGGGTA	
*Babesia major*	CCTeta	Bmaj_F	CACTGGTGCGCTGATCCAA	75
		Bmaj_R	TCCTCGAAGCATCCACATGTT	
		Bmaj_P	AACACTGTCAACGGCATAAGCACCGAT	
*Theileria parva*	18S rRNA	Tpar_F	GAGTATCAATTGGAGGGCAAG	173
		Tpar_R	CAGACAAAGCGAACTCCGTC	
		Tpar_P	AAATAAGCCACATGCAGAGACCCCGAA	
*Theileria taurotragi*	18S rRNA	Ttau_F	GGTCTTGGCACGTGGCTTTT	80
		Ttau_R	AGCCTGCTTTGAGCACTCTAA	
		Ttau_P	TTCGGACGGTTCGCTGTCTGGATGT	

### Sequence analysis and phylogenic tree analysis

For phylogenetic analysis, the 28S sequence data obtained via NGS (Table 2) were aligned and subsequently compared with parasitic species data from GenBank using the phyml v2.4.4 software [13], [14]. Distance matrices were calculated using the General time reversible (GTR) model and bootstrap analysis was performed with 1000 replications [15]. *Plasmodium falciparum*, a close apicomplexa was used as an out-group.

**Table 2:** Illumina sequencing of parasitic sequences identified within *I. ricinus* ticks and sequence read number matched to reference genomes.

Suspected genus	Closest species	Contigs	% identity (e-value)	Target gene	Contig length	Read number
*Babesia* spp.	*Babesia divergens*	*131510*	100% (1e-55)	18S rRNA	181	254
		*144996*	96% (2e-110)	18S rRNA	318	3351
		*154119*	100% (2e-13)	18S rRNA	101	97
	*Babesia microti*	*107742*	100% (1e-31)	18S rRNA	137	271
		*108532*	99% (5e-70)	18S rRNA	222	1251
		*120900*	100% (6e-14)	18S rRNA	102	1896
		*108977*	97% (2e-100)	unknown	233	336
		*153009*	100% (1e-22)	unknown	130	852
	*Babesia* sp. EU1	*112965*	100% (3e-26)	18S rRNA	195	918
		*150098*	100% (2e-79)	18S rRNA	224	480
		*172249*	100% (4e-56)	18S rRNA	213	76
		*114792*	100% (3e-27)	18S rRNA	129	366
	*Babesia major*	*145999*	97% (3e-93)	28S rRNA	270	2351
*Theileria* spp.	*Theileria parva*	*127324*	94% (4e-34)	28S rRNA	163	237
		*131568*	96% (1e-39)	28S rRNA	188	149
		*164638*	97% (2e-29)	28S rRNA	139	149
	*Theileria taurotragi*	*110157*	97% (4e-87)	28S rRNA	197	1216

## Results and Discussion

To identify known, novel or unexpected parasites carried by ticks in France, *I. ricinus* were collected in Eastern France, a wooded region with high tick abundance. Using NGS techniques, 17 contigs were selected following the criteria previously described and are presented in Table 2. Parasites from two distinct genera were identified: *Babesia* spp. (13 sequences), and possibly *Theileria* spp. (4 sequences). Other eukaryotic sequences with significant identity to sequences present in the databank corresponded mainly to fungi (*Ascomycota*) and are not presented here.

### Parasites from the *Babesia* genus

Three zoonotic *Babesia* species, *B. divergens*, *B. microti* and *Babesia* sp. EU1 were identified in *I. ricinus*, in addition to *B. major*, a parasite that only infects cattle. Transovarial transmission within ticks is characteristic of *Babesia* spp., implying that ticks constitute a real parasite reservoir in the field.

#### *B. divergens*

Following our criteria, three sequences related to *B. divergens* 18SrRNA were identified via NGS sequencing (Table 2), but no products were obtained after qPCR with specific primers aimed to amplify the *hsp*70 gene specific to this species. This result suggests that the parasite exists in small numbers, which is under the PCR threshold of detection but detectable with NGS due to the high number of transcripts corresponding to the 18S rRNA gene. *B. divergens* is a bovine parasite transmitted by *I. ricinus*, and is thought to be responsible for most cases of human Babesiosis in Europe, and especially, but not exclusively, in splenectomized patients [2], [16]. This parasite is the most widespread and pathogenic *Babesia* species infecting cattle in Northern temperate areas [17]. Traditionally, *B. divergens* has had a high serological prevalence in cattle from Western or Central France [17]. The discovery of this parasite in Eastern France may suggests that its geographical distribution is increasing, even within forested areas without cattle farms, which would require the existence of (an as-yet unidentified) reservoir hosts other than cattle. Further epidemiological studies are then now required in order to confirm that the parasite is now established in the studied area.

#### *Babesia* sp. EU1

NGS analysis identified four contigs related to the *Babesia* sp. EU1 18S rRNA encoding gene (Table 2) and the DNA presence of this species was confirmed by qPCR. This species, implicated in human cases of Babesiosis in Europe [18], [19], seems to phylogenetically lie in a sister group with *B. divergens* [18] in fact some serological cross-reactivity between *B. divergens* and *Babesia* sp. EU1 has been reported [20]. Roe deer were strongly suspected to be the wild reservoir of this parasite [6], [21] and its transmission by *I. ricinus* was validated both *in vivo* [6], [22] and *in vitro* [5]. In addition, *Babesia* sp. EU1 has been identified in *I. ricinus* in several European countries including Slovenia [23], Switzerland [24], the Netherlands [25], Poland [26], Italy [27], Belgium [28] and France [6], [8], demonstrating a wide geographical spread across the continent. Increasing reports of *Babesia* sp. EU1 in ticks and wild ruminants makes this parasite an excellent candidate for the emergence of a new zoonotic tick-borne disease.

#### *B. microti*

Five sequences related to *B. microti* 18S rRNA gene were identified following NGS analysis (Table 2) but were also not confirmed by qPCR aimed at amplifying the CCTeta gene. This result represents the first identification of this species in ticks from France. However, it is not surprising that this particular *Babesia* species was detected in wooded areas, as this rodent parasite is known to be transmitted by *I. ricinus*, and now seems to be widely established in Europe. Indeed, *B. microti* has been identified in *I. ricinus* in several European countries such as Switzerland [29], Poland [30], Slovenia [31], Germany [32], the Netherlands [25], [33] and Belgium [28]. To date, only two cases of human Babesiosis caused by this parasite have been reported in Europe [34], [35], but its zoonotic impact is well known in the United States [36]. Furthermore, autochthonous cases of *B. microti* infections have been diagnosed in Taiwan and Japan [37], [38], emphasizing the increasingly greater world distribution of this parasite.

#### *B. major*

NGS analysis revealed one contig with 97% similarity to the *B. major* 18S rRNA gene (Table 2). Despite a high number of reads obtained (2351), we also failed to confirm the presence of *B. major* DNA by qPCR for the CCTeta gene. *B. major* is a temperate-zone species able to infect cattle with lower pathogenicity than *B. divergens*, and has a far more limited geographical distribution which is linked to its tick vector, Haemaphysalis *punctata. Whether finding RNA from this parasite in *I. ricinus* ticks is epidemiologically relevant, needs to be clarified with additional laboratory competency experiments. Indeed, even if no human cases have been reported for this parasite, its occurrence in *I. ricinus* ([39] and this study), a tick which frequently bites humans, as well as the fact that several *Babesia* species have been shown to have wider vertebrate host ranges than previously thought [39], [40], may justify surveillance of this parasite.*

### Parasites from the *Theileria* genus

Following our selection criteria, four sequences were identified as belonging to the *Theileria* genera (Table 2). Three were most closely related to *T. parva* with 94–97% 18S rRNA identity, but with relatively low e-values and numbers of associated reads (535 in total). The presence of *T. parva* DNA was however confirmed by qPCR also based on the 18S rRNA sequence. The fourth sequence appeared to be related to *T. taurotragi* (97% 18S rRNA identity) with higher e-values and read numbers (1216), but no amplification could be obtained after qPCR with specific primers for the 18S rRNA encoding gene. These results indicate that some related *Theileria* species, but different from *T. parva* or *T. taurotragi*, are detected in *I. ricinus*. *Rhipicephalus appendiculatus* is the most common vector for *T. parva* and *T. taurotragi*, but other *Rhipicephalus* species can also transmit these organisms, implying flexible vector specificity. Both species occur in Africa, where *T. parva* mainly infects cattle, whereas *T. taurotragi* was found to have a wider host range [41].

**Figure 1:**
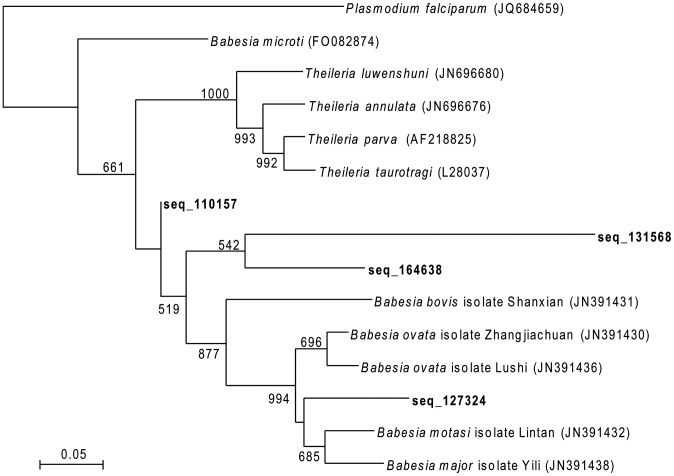
Maximum likelihood phylogenetic tree of partial 28S rRNA parasites sequences. GenBank accession numbers are given in parentheses. NGS Sequences are indicated in bold. Numbers represent bootstrap values (%) based on 1000 replications. Only bootstrap values higher than 500 are reported.

Phylogenetic analysis based on 28S NGS sequence data indicated that all four ambiguous sequences (127324, 131568, 164638 and 110157) seemed to belong to distinct and novel apicomplexa species (Figure 1). Only one sequence (110157), with the highest probability and read number, was confirmed to be related to a *Theileria* species. The other three seem to belong to *Babesia* species. However, considering that very few complete parasite genome sequences are available, parasite identification is mainly performed on the basis of 18S or 28S rDNA sequence analysis. These are the most highly represented parasitic sequences in GenBank, but are not the most informative in terms of species assignation. Moreover, this preliminary analysis was performed with short sequences (139–197 bp), which are not located at the same region within the 28S rDNA, therefore no definite species can be identified. Thus, further investigations are now required to clarify whether the identification of new *Theileria* or *Babesia* species in France, similar to tropical species, actually corresponds to an expanded geographical distribution of these species, and whether they have a potential pathogenic effect in mammals. Unfortunately, the absence of tick-borne parasite genome data causes difficulties in realizing such phylogenetic studies. However, in spite of the low level of robustness of theses phylogenetic analyses, our results are confirmed by other studies, in particular those demonstrating that the *B. microti* group is entirely divergent from either *Babesia* sensu stricto or *Theileria* species [42].

### Conclusion

The inventory of parasitic RNA content in *I. ricinus* performed by NGS revealed the presence of expected viable parasites belonging to the *Babesia* genus, some of them being identified in France for the first time. However, the epidemiological relevance of these results must of course be interpreted with caution. Unfortunately, complete genomic data on tick-borne parasites is scarce, likely due to large genome complexity compared to the relatively small number of research teams in this field. In addition, their small genome size and the strong inter-species conservation of available sequences (essentially 18S rRNA), does not permit clear species identification. Moreover, unknown species with too distant alignment and the fewest database sequences could not be identified in this context. The increased number of sequences relative to tick-borne parasites in data banks should facilitate an increase in the power of NGS techniques to detect tick-borne parasites in the future. In addition, detecting pathogenic RNA within ticks does not imply that these pathogens are actually transmitted by this arthropod. Therefore competence and epidemiological studies are also required in order to verify whether *I. ricinus* is implicated in the transmission of those tick-borne diseases which are present or emerging in France. And finally, further studies are also required to confirm whether the unexpected *Theileria* species detected here is actually novel, and whether the detection of parasitic species similar to other tropical species in France, corresponds to increasing geographical species distribution.
